# OF-Pelvis classification of osteoporotic sacral and pelvic ring fractures

**DOI:** 10.1186/s12891-021-04882-6

**Published:** 2021-11-29

**Authors:** Bernhard W. Ullrich, Klaus J. Schnake, Ulrich J. A. Spiegl, Philipp Schenk, Thomas Mendel, Lars Behr, Philipp Bula, Laura B. Flücht, Alexander Franck, Erol Gercek, Sebastian Grüninger, Philipp Hartung, Cornelius Jacobs, Sebastian Katscher, Friederike Klauke, Katja Liepold, Christian W. Müller, Michael Müller, Georg Osterhoff, Axel Partenheimer, Stefan Piltz, Marion Riehle, Daniel Sauer, Max Joseph Scheyerer, Philipp Schleicher, Gregor Schmeiser, René Schmidt, Matti Scholz, Holger Siekmann, Kai Sprengel, Dietrich Stoevesandt, Akhil Verheyden, Volker Zimmermann, the Spine Section of the German Society for Orthopaedics and Trauma

**Affiliations:** 1 Department of Trauma, Hand and Reconstructive Surgery, Jena University Hospital, Friedrich Schiller University Jena, 07747 Jena, Germany; 2 Department of Trauma and Reconstructive Surgery, BG Klinikum Bergmannstrost Halle, Halle, Germany; 3 Center for Spinal and Scoliosis Surgery, Waldkrankenhaus Erlangen, Erlangen, Germany; 4 Department of Orthopedics and Traumatology, Paracelsus Private Medical University Nuremberg, Nuremberg, Germany; 5 Department of Orthopaedics, Trauma Surgery, and Plastic Surgery, University of Leipzig, Leipzig, Germany; 6 Department of Science, Research and Education, BG Klinikum Bergmannstrost Halle, Halle, Germany; 7 Sana Klinikum Borna, Department of Spine Surgery and Neurotraumatology, Borna, Germany; 8 Departement for Trauma- and Reconstructive Surgery, Orthopedics, Hand- and Plastic Surgery, General Hospital Gütersloh, Reckenberger Straße 19, 33332 Gütersloh, Germany; 9 Department of Trauma Surgery and Orthopedics, Regiomed Clinical Center Coburg, Ketschendorfer Str. 33, 96450 Coburg, Germany; 10 Center for Trauma Surgery and Orthopedics, Helios University Hospital Wuppertal, University Witten/Herdecke, 42285 Wuppertal, Germany; 11 Spine Center, St. Josefs-Hospital Wiesbaden GmbH, Wiesbaden, Germany; 12 Center for Spine Surgery, St. Remigius Hospital Leverkusen, Leverkusen, Germany; 13 Department of Spine Surgery, Thuringia Clinic “Georgius Agricola” Saalfeld, Teaching Hospital of the University of Jena, Saalfeld, Germany; 14 Department of Orthopaedics and Trauma, Asklepios Klinik Wandsbek, Hamburg and Hannover Medical School (MHH), Hannover, Germany; 15 Department of Orthopedic and Trauma Surgery, University Medical Center Schleswig-Holstein, Campus Kiel, Germany; 16 Spine & Sport – Trauma / Ortopaedics / Sports Medicine, Herrenhäuser Kirchweg 38, 30167 Hannover, Germany; 17 Department of Trauma, Reconstructive Surgery and Orthopedics, RKH Krankenhaus Bietigheim, Bietigheim, Germany; 18 Department of Spine Surgery, Schoen-Clinic München Harlaching, Harlachingerstrasse, 51 Munich, Germany; 19 Department of Orthopaedic and Traumatology, University of Cologne, Faculty of Medicine and University Hospital Cologne, Joseph-Stelzmann-Straße 24, 50931 Cologne, Germany; 20 Center for Spinal Surgery and Neurotraumatology, Berufsgenossenschaftliche Unfallklinik Frankfurt am Main gGmbH, DE-60389 Frankfurt am Main, Germany; 21 Department of Spine Surgery, Schoen-Clinic Hamburg Eilbek, Dehnhaide 120, 22081 Hamburg, Germany; 22 Department of Orthopedics and Traumatology Alb Fils Hospital Eichertstr, 3 73035 Göppingen, Germany; 23 Clinic of Trauma-, Hand- and Reconstruction Surgery, AMEOS-Clinic Halberstadt, Gleimstr. 5, 38820 Halberstadt, Germany; 24 Department of Trauma und Interdisciplinary Spine Center, University Hospital Zurich (USZ), University of Zurich (UZH), Raemistrasse 100, 8091 Zurich, Switzerland; 25 University Clinic and Poliklinik of Radiology, Martin-Luther-University, Ernst-Grube-Str. 40, 06120 Halle (Saale), Germany; 26 Clinic for Trauma, Orthopedic and Spine Surgery, Ortenauklinikum Lahr-Ettenheim, 77933 Lahr, Germany; 27 Department of Trauma and Orthopedic Surgery, Klinikum Traunstein, Germany

**Keywords:** Sacral, Pelvic ring, Fracture, Osteoporosis, Classification, Consensus development, Reliability

## Abstract

**Objectives:**

Osteoporotic fractures of the pelvis (OFP) are an increasing issue in orthopedics. Current classification systems (CS) are mostly CT-based and complex and offer only moderate to substantial inter-rater reliability (interRR) and intra-rater reliability (intraRR). MRI is thus gaining importance as a complement.

This study aimed to develop a simple and reliable CT- and MRI-based CS for OFP.

**Methods:**

A structured iterative procedure was conducted to reach a consensus among German-speaking spinal and pelvic trauma experts over 5 years. As a result, the proposed OF-Pelvis CS was developed. To assess its reliability, 28 experienced trauma and orthopedic surgeons categorized 25 anonymized cases using X-ray, CT, and MRI scans twice via online surveys. A period of 4 weeks separated the completion of the first from the second survey, and the cases were presented in an altered order. While 13 of the raters were also involved in developing the CS (developing raters (DR)), 15 user raters (UR) were not deeply involved in the development process.

To assess the interRR of the OF-Pelvis categories, Fleiss’ kappa (κ_F_) was calculated for each survey. The intraRR for both surveys was calculated for each rater using Kendall’s tau (τ_K_). The presence of a modifier was calculated with κ_F_ for interRR and Cohen’s kappa (κ_C_) for intraRR.

**Results:**

The OF-Pelvis consists of five subgroups and three modifiers. Instability increases from subgroups 1 (OF1) to 5 (OF5) and by a given modifier. The three modifiers can be assigned alone or in combination.

In both surveys, the interRR for subgroups was substantial: κ_F_ = 0.764 (Survey 1) and κ_F_ = 0.790 (Survey 2). The interRR of the DR and UR was nearly on par (κ_F_ Survey 1/Survey 2: DR 0.776/0.813; UR 0.748/0.766). The agreement for each of the five subgroups was also strong (κ_F_ min.–max. Survey 1/Survey 2: 0.708–0.827/0.747–0.852). The existence of at least one modifier was rated with substantial agreement (κ_F_ Survey 1/Survey 2: 0.646/0.629).

The intraRR for subgroups showed almost perfect agreement (τ_K_ = 0.894, DR: τ_K_ = 0.901, UR: τ_K_ = 0.889). The modifier had an intraRR of κ_C_ = 0.684 (DR: κ_C_ = 0.723, UR: κ_C_ = 0.651), which is also considered substantial.

**Conclusion:**

The OF-Pelvis is a reliable tool to categorize OFP with substantial interRR and almost perfect intraRR. The similar reliabilities between experienced DRs and URs demonstrate that the training status of the user is not important. However, it may be a reliable basis for an indication of the treatment score.

## Introduction

In 1982, Lourie first described sacral insufficiency/fragility fractures as a “spontaneous osteoporotic fracture of the sacrum: an unrecognized syndrome of the elderly” [1]. Today osteoporotic fractures of the pelvis (OFP) are an increasing issue in orthopaedics with increasing incidence, relevant health care costs, high morbidity and high mortality [2].

Several classification systems (CS) focusing on various aspects have been developed over time. However, the established CSs did not describe the special issues of OFP well until the “comprehensive classification of fragility fractures of the pelvic ring” (FFP) was published in 2013 to deal specifically with OFP [3]. Another CS that Bakker et al. published in 2018 considers only osteoporotic sacral fractures; hence, it is not comprehensive for the entire pelvic ring [4]. In contrast, the 2019 published alphanumeric CS (ANC), like the FFP, considers the sacrum and the entire pelvic ring while the AOSpine sacral CS (AOSpine SCS) focuses mainly on sacral fracture patterns. Anterior pelvic ring injuries and sacroiliac joint lesions are taken into account in this CS via modifiers. Insufficiency fractures are summarized under C0 and not further differentiated [5].

All of the above CSs are based solely on CT findings [3, 5, 6]. Only Bakker et al. used the combinations of CT and MRI in four of the 130 described cases [4]. In 2015, Nuchtern et al. showed that 17% of the posterior pelvic ring injuries were missed when only CT was used in an osteoporotic cohort [7]. Mendel et al. also found that in a series of 78 bilateral fragility fractures of the sacrum, contralateral fracture involvement could only be detected via MRI in 17 cases (22%) [8]. Nevertheless MRI findings are relevant and influence treatment decisions [9–11].

Considering the above-mentioned issues and limitations of the available CSs, efforts to develop a CS for OFP are worthwhile. Motivated by the successful development of the CS for osteoporotic thoracolumbar spinal fractures [12] the working group „Osteoporotic Fractures “(AG OF) of the spine section of the German Society for Orthopaedics and Trauma (DGOU) commenced the OF-Pelvis project.

The objective of this endeavor has been, first, to view osteoporotic fractures of the sacrum and pelvic ring as an entire entity. Second, CT and MRI findings should be considered, and the degree of instability should correlate to the classification categories. Finally, the new CS should be reliable and easy to use.

## Methods

The AG OF’s work followed “A Concept for the Validation of Fracture Classifications” which Audigé et al. published in 2005 [13]. In the first phase classification categories where defined through an iterative process of drafts and evaluations. In the second phase an agreement study among representative future users was performed. The third phase that Audigé introduced (a prospective clinical study assessing the usefulness of the CS) has not yet been performed.

### First phase

Consecutive meetings of the AG OF were inaugurated. Two meetings analyzing the state-of-knowledge were held in September and November of 2015. Pelvic researchers of the DGOU were invited to lecture during the meetings. The FFP as the current standalone CS for OFP [3] and the existing CSs for non-osteoporotic sacral and pelvic ring fractures and anatomical findings of the sacrum [14] were evaluated.

In subsequent meetings the AG OF performed an extensive literature review and discussed the findings in the context of the current classifications.

The first pre-evaluation evaluated the inter-rater reliability (interRR) of the first classification draft following seven subsequent meetings. The reasons for disagreements were examined and the classification draft was modified according to these insights. Two additional evaluations assessed the revised interim classification drafts. The actual classification draft was thus developed, and the first phase was finished in an iterative process involving 16 meetings with 10–20 participants (mean 13 ± 2).

### Second phase

The performance of this phase was orientated toward the evaluation of the reliability of the AOSpine thoracolumbar spine injury and sacral CSs [5, 15]. All members of the spine section of the DGOU, not only members of the AG OF, were invited to participate in two online surveys, which were conducted 4 weeks apart.

The surveys were conducted online using REDCap software (Version 6.5.2, Nashville, TN: Vanderbilt University). Cases were presented online to all participants. To ensure that the raters had an understanding of the CSs, they were instructed to read a written tutorial, which was sent 2 weeks before the first survey. Before completing the first and second surveys, the raters also watched a 10-min tutorial, followed by a trial run of three cases. All raters were asked to have an explanation sheet detailing the OF-Pelvis classification readily available during survey completion.

### Case selection and presentation

All developing members of the AG OF were invited to send cases of each category to the study center. Out of 120 anonymized cases, by a team of experts (B.U., K.S. and U.S.) 25 typical cases including all subgroups and modifiers were selected and a “gold standard” defined. These 25 cases were not evaluated in the development process.

For the evaluation DICOM data were prepared to give every case the same formal appearance, which consisted of key images (conventional radiograph of the pelvis as well as selected MRI and CT slices) and a video sequence with all axial, coronal and sagittal CT and MRI slices The evaluation was performed in two surveys with 4 weeks in between and altered order of cases in the second survey.

Twenty-eight orthopedic and trauma surgeons experienced with OFP evaluated the classification. Thirteen members of the AG-OF who had also participated in the development meetings were defined as developing raters (DR). Fifteen members of the spine section of the DGOU who where not involved substantially in the development process were defined as User Rater (UR).

### Statistics

Data were collected in Microsoft Excel sheets using REDCap and exported to SPSS (IBM SPSS Statistics for Windows, Version 27.0, Armonk, NY: IBM Corp) for statistical analysis. Initially Fleiss´ kappa (κ_F_) was used to analyze interRR for the OF Pelvis overall, as well as for the five subgroups. The total interRR was analyzed of the complete rater cohort, before being analyzed according to differentiated considerations for the DRs and URs. The interRR was calculated for both surveys. Kendall’s tau (τ_K_) was used to calculate the intra-rater reliability (intraRR) for the entire group of raters and for the DR and UR subgroups.

The interRR for identifying a modifier in general was analyzed using a dichotomous variable—whether a modifier was found in principle or not—for both surveys. Additionally, the interRR was calculated separately for each of the three modifiers across the entire rater cohort, as well as for the DR and UR subgroups. Here, Cohen’s kappa (κ_C_) was also employed to describe the intraRR for modifier detection in principle and for each modifier separately, for the entire rater cohort, and for the DR and UR subgroups, respectively.

In interpreting the κ_F_, τ_K_, and κ_C_ values, the Landis and Koch classification and interpretation criteria [16] were used to indicate agreement (slight: 0.01–0.20, fair: 0.21–0.40, moderate: 0.41–0.60, substantial: 0.61–0.80, and almost perfect: 0.81–1.00). The proportion of the raters in total agreement with the gold standard was calculated for each case. Hereafter, the mean of this proportion, which was calculated separately for the OF-Pelvis, its five subgroups, an identified modifier in principle, and the three separate modifiers, was used to indicate absolute agreement.

### Classification proposal

**Fig. 1:**
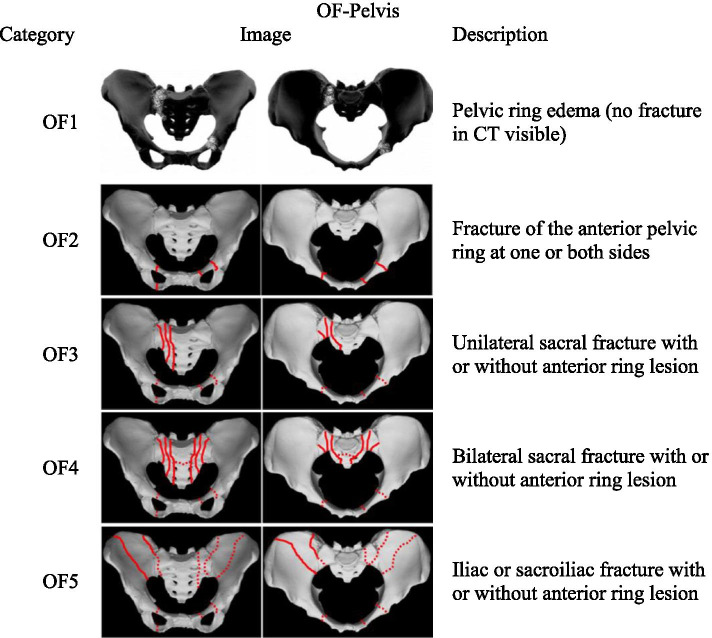
Localization of edema in OF1 and fracture localization in OF 2 – OF5. Continuous lines are variants of inevitable localizations for classification while broken lines are facultative localizations. Regarding edema detection needs MRI or Dual Energy CT OF 1 is presented in a different way than fracture lines in OF2-OF5

The developed OF-Pelvis, which consists of five subgroups and three modifiers, is depicted in Fig. 1. In general a fracture is defined by the coincidence of CT- and MRI-findings at the same localization. The case of edema detected in the pelvic ring via MRI without fracture signs in the CT is described as OF1. Fractures detectable via both CT and MRI are classified by subgroups OF2-OF5 depending on fracture localization. OF2 is a fracture of the anterior pelvic ring at one or both sides with uninjured posterior pelvic ring structures. OF3 is a unilateral sacral fracture and OF4 is a bilateral sacral fracture. An anterior ring lesion is facultative for both. With or without an anterior ring lesion, OF5 is an iliac or sacroiliac fracture that is highly unstable, due to the absence of fracture-spanning ligamentous structures in that fracture pattern (Fig. 1). So the degree of instability should increase from OF1 to OF5.

**Fig. 2:**
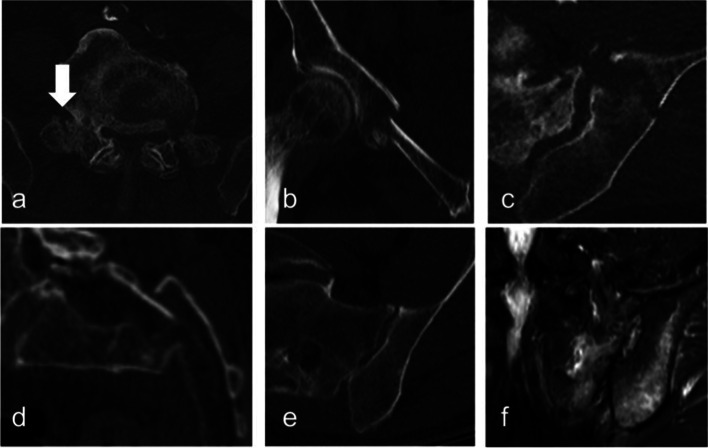
Three modifiers of the OF-Pelvis classifications system for osteoporotic sacral and pelvic ring fractures. Modifier can be assigned alone or in combination and shall indicate more severe injury a) Modifier 1 fracture of the L5 transverse process b-d) Displacement at any localization e) CT shows only a fracture of the sacral ala and f) the MRI reveals additional edema in the iliac bone without fracture evidence in the CT

The modifiers indicate a higher degree of instability than assumed according to the subgroup only. The importance of the iliolumbar ligaments for OFP is described in the literature [8, 17]. In respect to this the modifier M1 means an L5 transverse process fracture in the CT, which indicates involvement of the iliolumbar ligaments. Displacement is established as indicator for higher degree of instability e.g. in the FFP [3] and ANC [3] CS. So M2 means a displacement at any localization in each direction. Regarding that an edema can be detected in the absence of fracture line [7], the modifier M3 describes cases in which any edema is visible on the MRI at an additional localization to a confirmed fracture in the CT. The modifiers can be assigned alone or in combination (Fig. 2). They modifiers are not weighted by importance.

## Results

### Rater

Thirteen members of the AG-OF who had participated more than six times (7–15) at the 18 development meetings took part as DRs.

Fifteen members of the spine section of the DGOU and the AG OF who were not substantially involved in the development process (they had participated in less than six of the 18 meetings) and had not participated at all in the pre-evaluations took part in the evaluations as URs. All raters are experienced orthopedic and trauma surgeons with expertise in OFP.

### Reliability of the OF-pelvis subgroups

Table 1 presents the κ_F_ values for the interRR of the entire group of raters and for the DR and UR subgroups of the first and second surveys. The interRR of the OF-Pelvis showed higher values in the second survey (OF-Pelvis: 0.790, DR: 0.813, UR: 0.766). The lowest interRR was found among the URs in the first survey, but at 0.748, it was nevertheless strong. The confidence intervals also exhibited substantial agreement, with the lowest limit at 0.727.

**Table 1:** Fleiss’ kappa for inter-rater reliability of OF-pelvis subgroups 1 to 5 on the first and second surveys (Total: complete rater cohort, DR: developing rater, UR: user rater). For each survey the absolute agreement of the raters choice with the “gold standard” is given

		First survey			Second survey		
			0.95 CI	Absolute agreement		0.95 CI	Absolute agreement
		Kappa	(lower–upper limit)		Kappa	(lower–upper limit)	
Total	OF-Pelvis	0.764	(0.754–0.774)	89%	0.790	(0.780–0.800)	90%
	OF1	0.790	(0.769–0.810)	86%	0.809	(0.789–0.829)	87%
	OF2	0.815	(0.795–0.835)	94%	0.852	(0.832–0.872)	99%
	OF3	0.708	(0.688–0.729)	87%	0.750	(0.730–0.770)	89%
	OF4	0.717	(0.697–0.738)	90%	0.747	(0.727–0.768)	90%
	OF5	0.827	(0.807–0.847)	86%	0.808	(0.788–0.828)	86%
DR	OF-Pelvis	0.776	(0.754–0.799)	90%	0.813	(0.790–0.836)	91%
	OF1	0.772	(0.727–0.816)	87%	0.867	(0.823–0.912)	88%
	OF2	0.796	(0.751–0.840)	91%	0.831	(0.786–0.875)	98%
	OF3	0.727	(0.682–0.771)	88%	0.809	(0.765–0.854)	90%
	OF4	0.752	(0.708–0.797)	92%	0.775	(0.731–0.820)	94%
	OF5	0.866	(0.821–0.910)	88%	0.798	(0.754–0.842)	85%
UR	OF-Pelvis	0.748	(0.727–0.769)	88%	0.766	(0.746–0.785)	90%
	OF1	0.818	(0.777–0.859)	85%	0.759	(0.721–0.797)	85%
	OF2	0.827	(0.786–0.869)	97%	0.866	(0.828–0.904)	99%
	OF3	0.671	(0.630–0.712)	86%	0.704	(0.666–0.742)	89%
	OF4	0.684	(0.642–0.725)	89%	0.714	(0.676–0.752)	88%
	OF5	0.779	(0.738–0.820)	83%	0.805	(0.767–0.844)	87%

τ_K_ indicated a mean intraRR for the entire group of raters at 0.894 (0.95 CI: 0.862–0.926). The mean intraRR was 0.901 (0.95CI: 0.853–0.948) in the DR subgroup and 0.889 (0.95 CI: 0.840–0.938) in the UR subgroup (τ_K_).

In both the first and second surveys the absolute agreement was always exceeded 83% (the first survey OF5 for URs) and reached up to 99% for the entire group of 25 raters. (see Table 1).

### Modifiers

Table 2 presents the κ_F_ values for interRR among all raters, DR and UR, respectively, in identifying modifiers in each survey. Overall, the values, which ranged between 0.526 and 0.810, exhibited substantial agreement. Depending on the focus, the agreement can also be interpreted as moderate or even almost perfect. In general the interRR for the modifier was slightly below the interRR of the OF-Pelvis itself. Neither survey demonstrated a clear tendency toward better reliability.

**Table 2:** Fleiss’ Kappa for inter-rater Reliability in detecting a modifier in principle and in detecting the separate modifiers M1, M2 and M3 in the first and second surveys (Total: complete rater cohort, DR: developing raters, UR: user raters). For each survey, the absolute agreement of the raters´ choices with the “gold standard” is given

		1st survey			2st survey		
			0.95 CI	absolute agreement		0.95 CI	absolute agreement
	Modifier	kappa	(lower-upper limit)		kappa	(lower-upper limit)	
total	in principle	0.646	(0.625–0.667)	89%	0.629	(0.608–0.650)	90%
	M1	0.756	(0.736–0.776)	95%	0.777	(0.757–0.797)	95%
	M2	0.662	(0.642–0.682)	89%	0.569	(0.549–0.590)	86%
	M3	0.549	(0.529–0.569)	87%	0.602	(0.581–0.622)	88%
DR	in principle	0.667	(0.623–0.712)	89%	0.702	(0.657–0.746)	91%
	M1	0.810	(0.766–0.854)	96%	0.748	(0.703–0.792)	95%
	M2	0.718	(0.673–0.762)	90%	0.614	(0.570–0.659)	88%
	M3	0.568	(0.524–0.613)	88%	0.603	(0.559–0.648)	88%
UR	in principle	0.617	(0.576–0.658)	88%	0.565	(0.524–0.606)	89%
	M1	0.712	(0.674–0.750)	94%	0.803	(0.765–0.841)	96%
	M2	0.618	(0.579–0.656)	87%	0.532	(0.493–0.570)	85%
	M3	0.526	(0.487–0.564)	86%	0.608	(0.569–0.646)	88%

The intraRR showed a κ_C_ value of 0.684 for the detection of a modifier in principle (Table 3). Compared to modifiers M2 and M3, the modifier M1 showed the highest reliability across the entire group and in the DR and UR subgroups. The modifier M3 showed, in general, the lowest intraRR (0.664 across the entire rater group, 0.703 in the DR subgroup, and 0.630 in the UR subgroup). With the overlapping confidence intervals, the intraRR for the DR and UR subgroups were on par, although the URs showed a slight tendency toward lower values.

**Table 3:** Cohens Kappa for intra-rater reliability in detecting a modifier in principle and in detecting the separate modifiers M1, M2 and M3 in the first and second surveys (Total: entire rater cohort, DR: developing raters, UR: user raters)

			0.95 CI
	Modifier	Kappa	(lower–upper limit)
Total	In principle	0.684	(0.585–0.784)
	M1	0.777	(0.714–0.840)
	M2	0.720	(0.657–0.784)
	M3	0.664	(0.584–0.743)
DR	In principle	0.723	(0.611–0.835)
	M1	0.781	(0.669–0.894)
	M2	0.737	(0.668–0.806)
	M3	0.703	(0.588–0.818)
UR	In principle	0.651	(0.481–0.821)
	M1	0.773	(0.693–0.852)
	M2	0.706	(0.595–0.817)
	M3	0.630	(0.509–0.751)

The absolute agreement of the raters with the “gold standard” was very high with values between 85 and 96% (Table 2).

## Discussion

Several CSs [4] for OFP have been developed. The main drawbacks of the existing CSs include a lack of comprehensiveness, excessive complexity and/or low reliability. The proposed OF-Pelvis CS offers a comprehensive tool with a rather small numbers of subgroups and modifiers, but a substantial up to almost perfect interRR and intra RR. The ease of use is reflected in the small difference in reliability between the DR and UR subgroups.

### Classification of the whole pelvis

The FFP, ANC, and OF-Pelvis consider osteoporotic fractures of the sacrum and pelvic ring as an entire entity. In contrast the AOSpine SCS classifies the anterior lesion with only one modifier and does not focus on osteoporotic fractures. Aiming to classify fractures according to the risk of cement leakage following sacroplasty, Bakker et al. [4] considered only the sacrum. It is advisable however, to examine the entire pelvic ring and not the sacrum alone because the majority of OFPs show combined fracture patterns of sacral and anterior lesions [3, 18].

### Complexity and comprehensiveness

The OF-Pelvis consists of five subgroups and three modifiers, for a total of eight items only. Meanwhile the FFP contains four major categories with two to three subcategories each for a total of 11 groups [3]. The ANC includes three fracture types and three groups for two of the three types. Three subtypes are given for Group 1 and six subtypes each are given for group 2 and 3. Thus, a total of 31 fracture subtypes are possible [6]. The AOSpine SCS follows the AO principles with A, B and C indicating fracture severity and three subgroups, for a total of 10 types. It is clearly structured but is not applicable to OFPs which are summarized in one subgroup (C0) only without further differentiation [5]. In relation to the other above-mentioned CSs, the OF-Pelvis includes only five subgroups. The potential disadvantage of this simplicity is a lack of comprehensiveness.

From clinical and scientific perspectives, the requirements for a CS differ. While the clinical perspective demands an intuitive, easy-to-use, and clinically valuable tool, the scientific perspective prioritizes CSs that are able to describe almost any potential fracture pattern.

The AG OF decided to address the classification of OFP from the clinical perspective. Although this decision meant sacrificing the ability of the CS to describe a transverse fracture component of the sacrum, such as interconnecting bilateral fracture lines, in detail (such a case is summarized with OF4), this sacrifice was deemed acceptable because the impact of the missing information on the decision to pursue conservative versus surgical treatment is unclear. Furthermore, efforts to construct a CS capable of providing categories clearly aligned with surgical procedures to address each fracture type are limited by the lack of evidence for widely varying surgical treatment strategies for OFP [10, 19–21].

For the general decision regarding surgery versus conservative treatment, the development of a clinical score in addition to the OF-Pelvis is planned.

### The role of MRI

In 2008, Cabarrus et al. demonstrated the value of MRI for detecting OFP in relation to CT [22]. A review in 2010 by Lyders et al. underlined these findings [23]. A working group of pelvic surgeons subsequently illustrated the importance of the MRI for detecting pelvic injuries especially in geriatric patients [7, 24]. As a logical consequence it seems to be necessary to consider MRI-findings in the classification of OFP. A unique feature of the OF Pelvis in relation to the FFP, ANC, AOSpine SCS and Bakker et al. ´s classification scheme is the inclusion of MRI findings in the CS. From this perspective, the OF Pelvis is the most comprehensive CS available to date. Palm et al. found that dual-energy CT was able to detect bone edema in the pelvic ring with the same sensitivity and specificity as MRI at the pelvic ring [25]. Under the OF-Pelvis CS, edema detected with dual-energy CT in the absence of fracture lines at the same localization can be classified—like MRI detected bone edema—as OF1 or M3, respectively. If no MRI is present or available e.g. due to medical contraindications the OF-Pelvis CS can be used with limitation to detect subgroup OF1 and modifier M3. To assess the clinical benefit of MRI further studies are necessary. The OF-Pelvis CS could be a useful tool for those studies.

### Reflection of the degree of instability via classification

The FFP’s major categories aim to reflect the degree of a fracture’s instability, which is suitable for most cases. However, the iliolumbar and sacroiliac ligaments are considered to stabilize the spinopelvic junction [8, 26], yet the FFP seems not to focus on this topic. Having raised some concerns regarding the reliability of the FFP in demonstrating the degree of instability [6], Krappinger et al. introduced the ANC as a solution. The structure of the ANC is quite clear. Fractures localized at the anterior pelvic region (Type A) exhibit the lowest degree of instability, followed by fractures localized at the posterior pelvic ring (Type P) and finally combined anterior-posterior (Type AP) injuries with the highest degree of instability. Groups describe the presence of a uni- or bilateral fracture and transverse fracture components. These fractures can be complete or incomplete. The role of the sacroiliac and iliolumbar ligaments is considered by subgroups 1–3. Thus, compared to the FFP and the OF-Pelvis, the superior capability of the ANC to describe the degree of instability in detail cannot be denied. The greater complexity induced by the ANC’s 31 types may reduce its acceptance and lead to lower interRR and intraRR if tested by more than 4 raters. The AO Spine SCS is not discussed here because it is not applicable to OFP, which it summarily categorizes under C0 subgroup. Bakker et al. finally defined only one type (C3 displaced sacral U-type) as unstable.

In the OF-Pelvis CS, instability increases from OF 1 to OF 5. The most stable pattern is bone edema without fracture signs on the CT (OF1). The instability then increases from OF2 (anterior fracture only) to the involvement of the sacrum (OF3 on one side and OF4 on both sides). OF 5 is considered to have the highest degree of instability, as it lacks stabilization from spanning ligamentous structures. The modifiers (M1-M3) indicate a more severe fracture. M1 (fracture of the L5 transverse process) indicates primary or secondary insufficiency of the iliolumbar ligament especially in the case of fracture progress, as Mendel et al. and Rommens et al. described [8, 17]. M2 (displacement at any localization) is a common feature for instability in the FFP, ANC, AOSpine SCS and Bakker et al. CS. M3 (edema at any additional localization) considers that CT is not sensitive enough to detect all alterations of the pelvic ring. Thus, it is possible to include the MRI findings into therapy planning, e. g. from the perspective of potential fracture progress [8, 17]. Further biomechanical and/or finite elements studies are necessary to investigate the correlation of degree of instability and OF 1–5 subgroups and the biomechanical impact of modifier M1–3 on stability.

### InterRR and intraRR and methods of their evaluation

Krappinger et al. published interRR and intraRR values of the FFP CS in 2019. Their study involved four raters (three orthopedic traumatologists with varying levels of experience and one radiologist) who were asked to classify 100 pelvic CT scans according to the FFP in two sessions. With kappa (κ) values of 0.42 to 0.59, interRR for the major groups of the FFP was moderate. Meanwhile, κ values of 0.68 to 0.72 indicated the FFP’s substantial intraRR. The interRR and intraRR for subgroups of the FFP, finally, were slight to moderate, with κ values of 0.10 to 0.52 and 0.29 to 0.66, respectively [18].

Another evaluation of the FFP CS was published in 2019. It engaged six experienced and six inexperienced surgeons. Sixty CT scans where evaluated to determine the intraRR and interRR and the percentage of agreement with the gold standard, which was defined by one surgeon whom the originator of the FFP trained. The study revealed moderate interRR (κ: 0.53) and intraRR (κ: 0.46) for the complete FFP CS and substantial interRR (κ: 0.61) and intraRR (κ: 0.60) for the major categories [27].

The study introducing the ANC demonstrated its moderate to substantial interRR and substantial overall intraRR, with κ values of 0.71 to 0.80. The authors used the same setting as in their above cited study [18]., finding that the ANC demonstrated overall reliability for classifying OFP’s comparable to that of the FFP [6].

Recently the AOSpine SCS was tested for its intraRR and interRR. For this purpose 18 surgeons reviewed 38 cases twice, with 4 weeks separating the two reviews. The AOSpine SCS showed excellent intraRR (κ: 0.83) and a substantial interRR (κ: 0.75) for severity as well as substantial intraRR (κ: 0.77) and moderate (κ: 0.64) interRR for all subtypes [5]. The “Bakker System” has not been evaluated to our knowledge to date [4].

The OF-Pelvis evaluation process followed the evaluation methodology of the AOSpine thoracolumbar CS [28, 29] and the AOSpine SCS [5]. The results of this study demonstrated substantial to almost perfect interRR (κ_F_ 0.684–0.866) and a almost perfect intraRR for the entire group of raters with a τ_K_ of 0.894 for the classification subgroups.

Despite a slight tendency toward higher reliability on the second survey, the interRR for the five subgroups exhibited strong uniformity overall. The modifiers - in general and in detail - showed mainly substantial interRR (κ_F_ 0.526–0.810) and constant substantial intraRR (κ_C_ 0.630–0.777). Thus, the agreement can be interpreted as substantial up to almost perfect, depending on the focus and the intraRR can be interpreted as almost perfect.

The system’s ease of use, moreover, is reflected in the small difference in reliability between the DR and UR subgroups. The OF-Pelvis in total reached excellent agreement with the gold standard of 89 and 90%.

Despite this supposed completeness and the possible increased variance associated with it, the OF-Pelvis CS achieves reliability measures that are superior to those of the previously established CSs. Due to the differing number of raters and cases as well the differing modes of case presentation a direct comparison of interRR and intraRR across the available CSs (e.g. FFP, ANS, AOSpine SCS) could be faulty. However, an evaluation of all considered CSs with the same raters, cases, and methods could provide reliable data for direct comparison.

To evaluate the clinical usefulness of OF Pelvis further research is necessary. The OF working group plans a Audigé’s phase three evaluation in which the OF Pelvis related treatment decisions are evaluated with clinical outcome assessments like e.g. Majeed Score [30] and possibly with a new prognostic pelvic injury outcome score which reveals higher interRR than e.g. Majeed score [31].

### Limitation

The comparability of this study’s results with those of other studies in that field is limited by the differing numbers of classified cases and participating raters. The differing methods employed to review the image data also hinder direct comparisons.

No conclusions are possible regarding the usefulness of the OF Pelvis without an as-yet unconducted third phase following Audigé.

Finally the selectivity between the user and developer rater subgroups is weak and all had certain knowledge of pelvic fractures, so this study’s assertions regarding users´ training statuses are limited.

## Conclusion

The OF-Pelvis is a simple a reliable classification system with substantial inter-rater reliability and almost perfect intra-rater reliability. Further, the OF-Pelvis considers in addition to the CT-data the MRI findings for classification, which makes it unique among existing CSs. However, the similar reliabilities between the developing rater and the using raters subgroups indicate the simple manageability of the CS’s. The OF-Pelvis may be a reliable basis for an indication of treatment score.

## Data Availability

The datasets used and analyzed with this study are available on reasonable request from the corresponding author.

## Abbreviations

AG-OF: Working group Osteoporotic Fractures of the Spine Section of the German Society for Orthopaedics and Trauma
ANC: Alphanumeric classification
AOSpine SCS: AOSpine sacral classification system
CS: Classification system
CT: Computed tomography
DGOU: German Society for Orthopaedics and Trauma
DR: Developing rater
FFP: Comprehensive classification of fragility fractures of the pelvic ring
interRR: Inter-rater Reliability
intraRR: Intra-rater reliability
MRI: Magnetic resonance imaging
OFP: Osteoporotic fractures of the pelvis
UR: User rater
τ_K_: Kendall’s tau
κ_C_: Cohen’s kappa
κ_F_: Fleiss’s kappa
